# Positive effects of physical activity in autism spectrum disorder: how influences behavior, metabolic disorder and gut microbiota

**DOI:** 10.3389/fpsyt.2023.1238797

**Published:** 2023-10-10

**Authors:** Annaluisa Ranieri, Cristina Mennitti, Noemi Falcone, Ilaria La Monica, Maria Rosaria Di Iorio, Lorella Tripodi, Alessandro Gentile, Maria Vitale, Raffaella Pero, Lucio Pastore, Valeria D’Argenio, Olga Scudiero, Barbara Lombardo

**Affiliations:** ^1^CEINGE-Biotecnologie Avanzate Franco Salvatore, Naples, Italy; ^2^Department of Molecular Medicine and Medical Biotechnologies, Federico II University, Naples, Italy; ^3^Task Force on Microbiome Studies, University of Naples Federico II, Naples, Italy; ^4^Department of Human Sciences and Quality of Life Promotion, San Raffaele Open University, Rome, Italy

**Keywords:** physical activity, autism spectrum disorder, gut microbiota, obesity, behavior

## Abstract

Autism spectrum disorder is a neurodevelopmental disorder characterized by social interactions and communication skills impairments that include intellectual disabilities, communication delays and self-injurious behaviors; often are present systemic comorbidities such as gastrointestinal disorders, obesity and cardiovascular disease. Moreover, in recent years has emerged a link between alterations in the intestinal microbiota and neurobehavioral symptoms in children with autism spectrum disorder. Recently, physical activity and exercise interventions are known to be beneficial for improving communication and social interaction and the composition of microbiota. In our review we intend to highlight how different types of sports can help to improve communication and social behaviors in children with autism and also show positive effects on gut microbiota composition.

## Introduction

**Figure 1:**
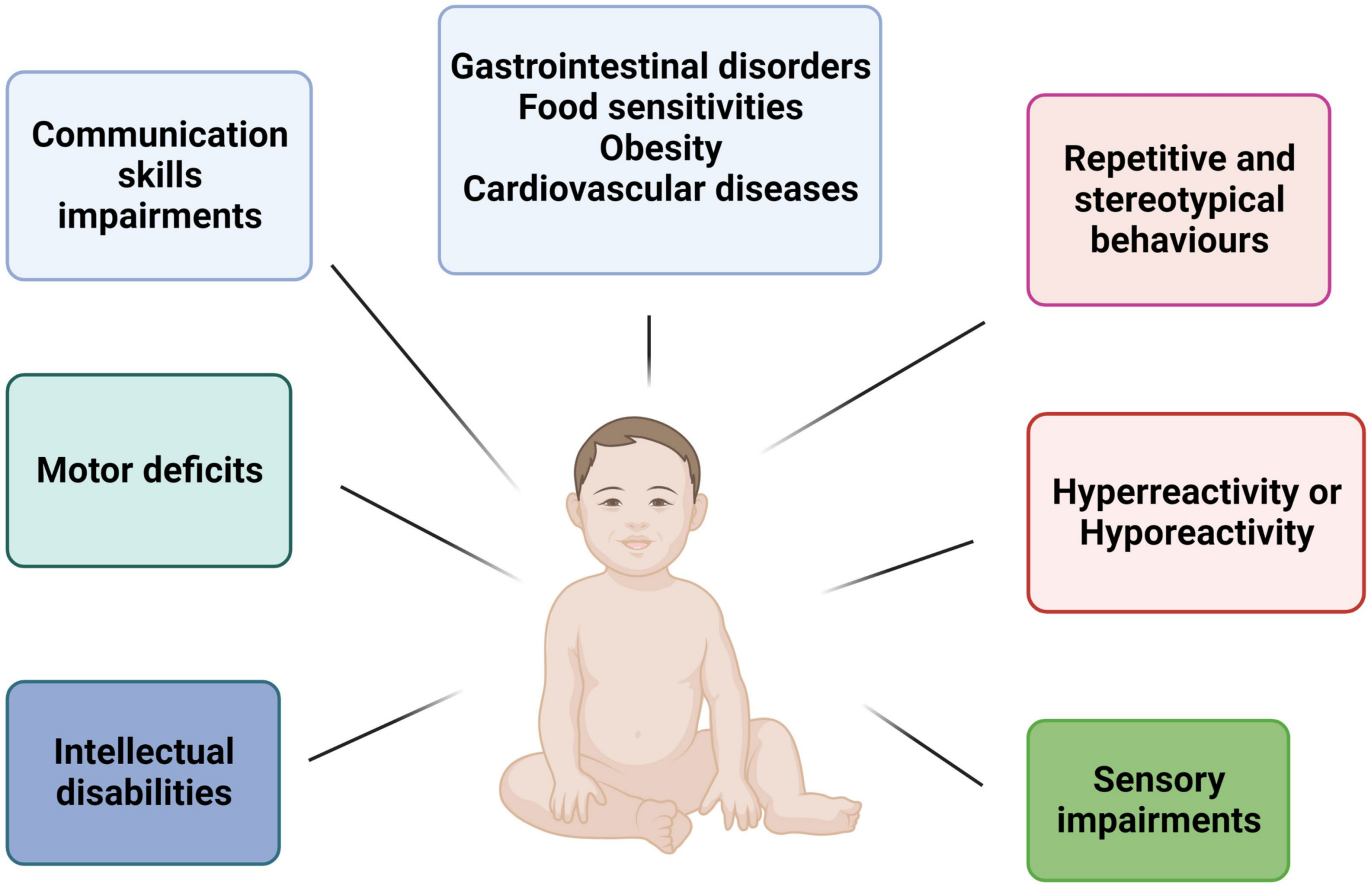
Schematic representation of main features in children with ASD. Children ASD-affected show several neurological and motor impairments such as intellectual disabilities, repetitive behaviors, communication impairments, and motor deficit. Furthermore, the main comorbidities are gastrointestinal disorders, food sensitivities, obesity, and cardiovascular diseases.

Autism spectrum disorder (ASD) is represented by a range of heterogeneous neurodevelopmental conditions characterized by impairments in social interactions and communication skills and the presence of repetitive and stereotypical behaviors and interests (1). In the Diagnostic and Statistical Manual of Mental Disorders (DSM-V) ASD includes classic autism, Asperger’s syndrome, Rett’s disorder, Childhood Disintegrative Disorder, and Pervasive Developmental Disorder – Not Otherwise Specified (PDD-NOS) (2). In recent years, the use of Array Comparative Genomic Hybridization (a-CGH) and whole exome/genome sequencing (WES/WGS) have enabled the molecular characterization of patients with ASD by identifying of copy number variations (CNVs) or single nucleotide variants (SNVs) in genes with a crucial role in the development of the central nervous system (CNS) (3–9). Autistic children exhibit impairments in social interaction, such as low eye contact, relational problems, and delays in verbal and nonverbal communication. Stereotypical behaviors and interests in children with ASD may include adherence to inflexible routines and motor stereotypies, such as hand flapping or body rocking (10). In addition, they can present cognitive and behavioral disorders that include attention difficulties, intellectual disability, anxiety, depression, aggression, and self-injurious behaviors (2). As for sensory disorders, autistic children can have difficulty in modulating tactile, auditory, visual, and vestibular inputs, with hypo-reactivity or hyper-reactivity to stimuli, and show comorbid systemic conditions such as gastrointestinal problems, food sensitivity, obesity, diabetes, and cardiovascular disease (10) (Figure 1). The Early Intensive Behavioral Intervention (EIBI) is a therapy that promotes learning, skill development, and behavior change through small steps that are easier to learn. In particular, in recent years, the possible role of the intestinal microbiota has emerged as a co-factor in the development of ASD, as a correct two-way communication between the intestine and the brain allows to regulate development and functions of the central nervous system. Indeed, several studies have demonstrated a connection between changes in the composition of the gut microbiota and the gastrointestinal and neurobehavioral symptoms found in autistic children (11). Moreover, in these subjects has been observed greater motor abnormalities, less coordination and postural impairments in static and dynamic balance. Thus, behavioral interventions are important in early ASD management to help individuals with autism to improve their quality of life in later years (1). The Early Intensive Behavioural Intervention (EIBI) is a therapy that promotes learning, skill development and behavior change through small steps that are easier to learn. In particular, in recent years, physical activity and exercise interventions are known to be beneficial for decreasing repetitive behaviors, improving communication and social interaction, reducing motor trouble and improving in working memory and meta-cognition (12, 13). The aim of this review was to show and analyze how different types of sports can help to correct stereotypical behaviors, improve motor skills, and overcome the difficulties of communication and social interaction in children with ASD. In particular, sport can act on sensory dysfunction situations, since the body can constantly receive different external stimuli through exercise. In fact, the eyes, ears, skin and nose are fully utilized during the exercise (14). Rich flows of sensory information in the brain activate the nervous system, promote the development of different areas of the nervous system, improve the sensory interaction capacity of the brain, improve motor skills, show positive effects on gut microbiota composition, and could help to enhance the quality of life for autistic patients with different sports such as swimming, minibasket, horse riding and karate (14).

## Obesity and related commorbidity in autistic children

Children with ASD are often exposed at an increased risk of developing overweight or obesity (15). Since 2019, it has been estimated that over 150 million children in the world are obese and that this will increase to 206 million by 2025 (16, 17). A meta-analysis by Sammels et al. revealed that the prevalence of obesity among children with ASD was 17%, and the children with ASD had a 58% greater risk of developing obesity compared with neurotypical children (18).

Knowledges on the relationship between obesity and autism are still uncertain. Although many of the risk factors for children with autism are likely the same as for the general pediatric population, there are unique factors implicated in the development of obesity in children with autism such as atypical eating patterns, physical inactivity, sleep disorders, psychopharmacological treatment, and genetics. In particular, the diet of autistic children is characterized by a preference for energy-dense foods, sugary drinks and snacks, with a rather limited consumption of vegetables (19). Moreover, children with ASD spend a lot of time in sedentary activities, such as watching TV, playing video games, and using social media (20).

The use of medications, as part of a treatment aimed at improving the management of problematic behavioral aspects of autism, can have undesirable effects. In recent years, typical and atypical antipsychotics are widely used in children with autism as an adjunct to antidepressants. Atypical antipsychotics, such as aripiprazole, clozapine, olanzapine, provide significant benefit in reducing the frequency of extrapyramidal symptoms. However, these second-generation antipsychotics (SGAs) are involved in weight gain (21). It has been seen that the use of SGAs in children is responsible for an increased risk of developing obesity and lipid abnormalities; these results are very significant since SGAs represent a first-line treatment in children with irritability associated with ASD (22).

Some children with ASD may also have a genetic predisposition to obesity: some microdeletions, such as 11p14.1 or 16p11.2, have been identified as responsible for this increased vulnerability (23, 24).

Autism has been observed to be associated with an increased risk of obesity-related conditions, such as type 1 and 2 diabetes mellitus, hypertension, dyslipidemia, and metabolic liver disease. Diabetes mellitus (DM) is a chronic disease characterized by hyperglycemia and disorders of carbohydrate, fat, and protein metabolism. There are three main forms of DM: Type 1 (T1DM), Type 2 (T1DM) and gestational diabetes mellitus (GDM). T1DM is an autoimmune pathology, dependent on an alteration of the immune system, which involves the destruction of insulin-producing beta cells; T2DM occurs as a result of insulin resistance with an alteration in the amount or function of insulin; finally, gestational diabetes is a metabolic disorder that occurs during pregnancy. Some researchers have hypothesized a possible link between autism and diabetes, although the underlying mechanisms are not yet fully understood. Several conditions such as Turner and Down syndromes appear to be responsible for a higher risk of T2DM in autistic individuals (25, 26), as well as the use of psychotropic drugs that lead to weight gain (20). Another possible mechanism is the elevated secretion of cytokines such as IL-1 and IL-6 in autistic subjects compared to healthy controls (27, 28), as in those with T1DM (29) and T2DM (30). Increased cytokine secretion and impaired immune function could contribute to pancreatic beta-cell destruction, resulting in the clinical expression of hyperglycemia. Regarding to GDM, possible factors connecting intrauterine hyperglycemia and the risk of autism in the unborn child may include oxidative stress (31), hypoxia (32), chronic inflammation (33), and epigenetics (34). In addition, diabetes mellitus gestational age is often accompanied by maternal obesity, which was associated with positive autism screening results in very preterm infants in a recent study (35).

Several studies have highlighted an altered cholesterol metabolism in patients with ASD (36, 37). At the brain level, cholesterol plays a fundamental role. It is in fact involved in numerous processes, such as neuronal development and function, synaptogenesis and myelination, formation of axons and dendrites. Furthermore, it is an important component of the membranes of neurons and is mainly located on the synaptic vesicles. Since plasma lipoproteins are unable to cross the blood brain barrier (BBB), cholesterol must be synthesized in the brain. Cholesterol is also responsible for the correct function of neurotransmitter, and an its impaired metabolism could cause brain dysfunction (38). On this basis, hypotheses concerning a possible relationship between autism and alterations of cholesterol metabolism have been advanced. A study conducted by Benachenhou et al. investigated changes in the lipid profile in a French-Canadian population with autism compared with a population of healthy individuals (36). The results showed that the autistic population has higher risk of developing hypocholesterolemia than healthy subjects and low total cholesterol levels have been associated with the development of symptoms of anxiety and depression.

## Physical activity and improvement of quality of life: swimming, karate, minibasket, and horse riding

**Figure 2:**
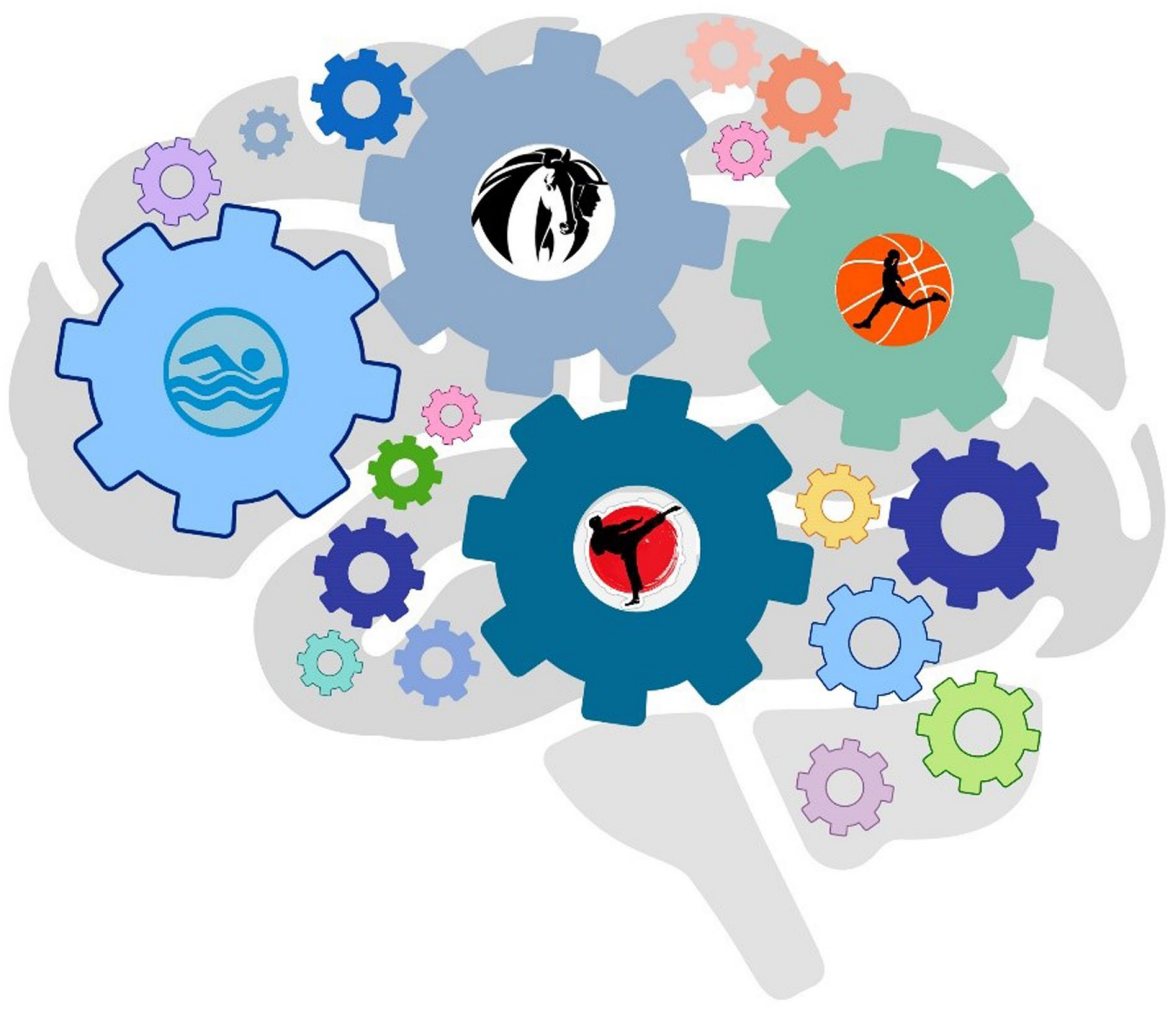
Physical activity ameliorates communication and social behaviors. Swimming, karate, minibasket, and horse riding in children with ASD stimulate different areas of the brain acting on cognitive, behavioral, and motor skills.

Sedentary lifestyle is typical for individual with intellectual disabilities. Individuals affected by autism are physically inactive both due to lack of motivation or interest in physical activity, and because participation requires motor and social skills that sometimes in autism syndrome lack. In the last decade, numerous studies have focused their attention on the effect of physical activity in individuals with autism, showing that specific physical activity induces improvements in behavior, health and motor skills (39, 40). Regular physical activity is a way to promote brain function and has many positive health effects. Exercise specifically made a significant difference in the symptomatology of ASD individuals and different physical activities can affect different biological mechanisms. However, it is still unclear exactly what neuronal processes contribute to the therapeutic effects of exercise (41). Some beneficial physical activities are described below (Figure 2).

### Swimming

Physical activity allows you to lead a healthy lifestyle, in fact, several studies have shown positive effects on motor skills and positive behavioral aspects in autistic children even with swimming. Swimming activities revealed to be efficacious to train psychomotor skills and increase adaptive behaviors in children with ASD (42). Several studies investigate the change in motor and behavioral aspects before and after a swimming program for autistic children (43–46). In particular, Battaglia et al. examine the efficacy of aquatic therapy on gross motor and social skills in three adolescents with ASD. The study showed that the aquatic training program was effective to enhance object control and locomotors skills in the analyzed subjects (43). This has also been proved by other studies that showed how exercises in water were effective to develop physical fitness and aquatic orientation and improve several aspects of gross motor proficiency, such as conditional (aerobic capacity, muscle strength, and speed) and coordination skills, in individuals with typical and atypical development (45, 47). As regards the behavioral and social skills, different studies showed that children with ASD had an increased eye contact, engaging in appropriate conversation with peers and instructors and following class rules and class routines (43, 47). Moreover, in Chien-Yu Pan’s study was reported a significant improvement in self-esteem, social interactions and motor skills was reported in children after 10 weeks of aquatic activities. It also highlighted a greater capacity for achievement and self-esteem of children with ASD (44). In fact, hydrotherapy represents a potential practice that would improve the relational, emotional and social integration aspects in individuals affected by ASD (48). In fact, during swimming, all muscles in the body are activated, afferent nerve stimuli to the CNS come from the proprioceptors of muscle fibers, and after processing, the CNS directs the impulses through the efferent fibers to the muscles. Therefore, swimming induces complex adaptations of the nervous system and promotes the transmission of neural impulses (49). However, there are no data in the literature explaining the biological mechanisms whose changes lead to improvements after a swimming program in children with ASD. However, a study conducted by Xu et al. on Shank3 knockout mice that had deficits in social memory, spatial memory, and object recognition showed that a swimming program improves learning and memory, hippocampal neural development, and plasticity in Shank3 knockout mice (50). In particular, several studies have reported that swimming improves spatial learning and memory in rats, this effect could be related to the action of brain-derived neurotrophic factor (BDNF) on brain plasticity (51, 52). In light of these studies, further research is needed to better understand the biological mechanisms that regulate the effect of swimming on children with ASD.

### Karate

Exercise not only improves physical condition but also reduces maladaptive behavioral patterns of people with ASD (53). In a study conducted by Bahrami, a karate-based training program was observed to significantly reduce communication difficulties in autistic children. Specifically, participants were (*N* = 30; 26 male) students aged 5–16 who had a previous diagnosis of ASD. These subjects were randomly divided into two groups: 15 ASD subjects who were assigned an exercise program and 15 ASD control subjects without an exercise program (54). Participants in the exercise condition were shown a sequence of rapid movements typical of martial arts in which athletes move in different directions in space to counter invisible opponents, to be reproduced in 56 sessions spread over 14 weeks. The results showed an improvement of the relational aspects in the autistic subjects involved in the karate sessions (54). Considering important new findings from psychology and neuroscience, it was proposed a neurobiological mechanism for these benefits. Several studies show that karate techniques develop postural motor functions, spatial orientations, and impact vestibular signaling patterns that challenge the hippocampus and enhance neurogenesis through the secretion of neurotrophins such as BDNF (54–56). One of the well-documented mechanisms includes increases in brain-derived neurotrophic factor (BDNF) levels. BDNF plays a pivotal role in neurological function, neurogenesis, survival regulation and neuronal differentiation, such as axonal pathways regulation, dendritic density, and synaptic plasticity. Additionally, the increase of BDNF was related to the improvements in memory and learning ability (57) and it was considered a key factor in a variety of neurodevelopmental disorders and neurodegenerative diseases. In the rat, brain BDNF promotes survival and sprouting of serotonergic axons and axonal growth of damaged serotonergic neurons. *In vitro* and *in vivo* studies support a regulatory role of BDNF in the survival and maturation of serotonergic neurons. BDNF has also been shown to modulate serotonergic neurotransmission *in vitro*, promote survival and differentiation of some cholinergic and dopaminergic neurons *in vitro*, and BDNF administration increases serotonin synthesis and/or turnover *in vivo* (55). The relation between BDNF alteration and neurological disorders suggest that BDNF could be consider a valid biomarker and therapeutic factor (58). Furthermore, serum BDNF levels was correlated with the severity of symptoms for young people with ASD. Several studies suggest BDNF as a mediator of the relationship between exercise and associated cognitive benefits, indeed, BDNF is upregulated up to 7 days after exercise (59) and the inhibition of BDNF receptors showed the reduction of the cognitive benefits associated with physical exercise (60). It has been reported that the karate practice induces the increase of the BDNF leading the amelioration of neuronal plasticity and recovering of the child’s neurological abilities (54).

### Minibasket

In autistic children, in the interpersonal relationships area, there may be attitudes of extreme isolation, closure and indifference in the face of human interactions and relationships with others. Among physical exercise programs, minibasket has a significant effect on improving integration and social communication (61). Minibasket, designed for children under 12, parallel to the physical and mental development of children and the development of executive functions, has the intrinsic characteristics of basketball, a sport that unites, relates, creates opportunities contact and stimulates new knowledge (61).

A study by Yang et al. showed how 12 weeks of minibasket, conducted on 30 autistic preschool children, significantly enhanced cognitive function, interpersonal skills and neural mechanism. Therefore, the improvement in the cognitive and social skills of preschool children with ASD could be attributed to the repetition in the movements of the teachers during the training sessions, which promote the social skills of the children while learning motor skills in minibasket (61). Indeed, the imitation process could improve the relational skills and social integration of these individuals (62). In minibasket, children must learn new movements and coordinate in team play, which requires a high level of control and cognition. As reported in literature the exercise can induce plasticity changes increasing functional connectivity of the executive control network between the right cerebellum and the left inferior frontal gyrus. These features were found in preschool children with ASD after 12 weeks of minibasket training program. Further studies have shown that the cerebellum is a key brain region involved in motor function such as coordination and balance (63, 64). In addition, it has been demonstrated, the amelioration of functions including memory, social interactions, and repetitive behavior after a 12 weeks in autistic children. These improvements probably are due to changes in BDNF leves in serum or plasma (65–67). More research is needed to study and clarify the neurobiological mechanisms that occurs in amelioration of physic and behavioral in children ASD affected.

### Horse riding

An interesting role for the develop of properly social interaction in autistic children, involved the therapy with animals and especially horses (68). Therapeutic riding was used to improve posture, balance, and mobility during the development, as well as establishing intimate interaction between subject and horse (69). In recent years, the interesting in equine therapy has grown, indeed, several studies have shown an improvement in social motivation and sensory sensitivity, as well as decreased inattention and distractibility (70). The study by Anderson et al. evaluates the effects of a 5 weeks equestrian therapy program on the social functioning of children or adolescents (*N* = 15) with ASD (71). The results confirmed that the riding increased empathy and reduced maladaptive behaviors, while specific adaptive behaviors such as socialization and communication were not affected by the intervention. In summary, the equine therapy does not change the entire behavior of the child but it can improve specific aspects of social function and reduce the maladaptive traits of ASD (72). Despite horse-riding does not change a child’s behavior, it can improve specific social function reducing the dysfunctional ASD traits, such as irritability and stereotyped behaviors (72). Although the mechanism is not completely clear, in some studies, it has been reported that the alteration of serotonin, dopamine, and GABA levels correlate with worsening of stereotypic behaviors (73). Therefore, rhythmic horse movements and prolonged contact with nature stimulates vestibular system that, in turn ameliorate the neurotransmitters production improving body coordination and emotional regulation avoiding extreme irritability and overreaction due to stress condition (73). Further study consistently examined the efficacy of equine assisted therapy (EAT) in improving adaptive and executive function in children with ASD (children attending EAT, *n* = 15, control group *n* = 13; inclusion criteria: IQ > 70). The results showed an improvement in social and executive functioning in the EAT group and a milder effect on motor skills (68, 74). However, the evidence for the positive effects of equine therapy on perceptual-motor, cognitive and functional skills is currently limited by unanimously positive results and many methodological weaknesses that make further research necessary (75).

## Gut microbial dysbiosis in ASD and potential role of physical exercise

**Figure 3:**
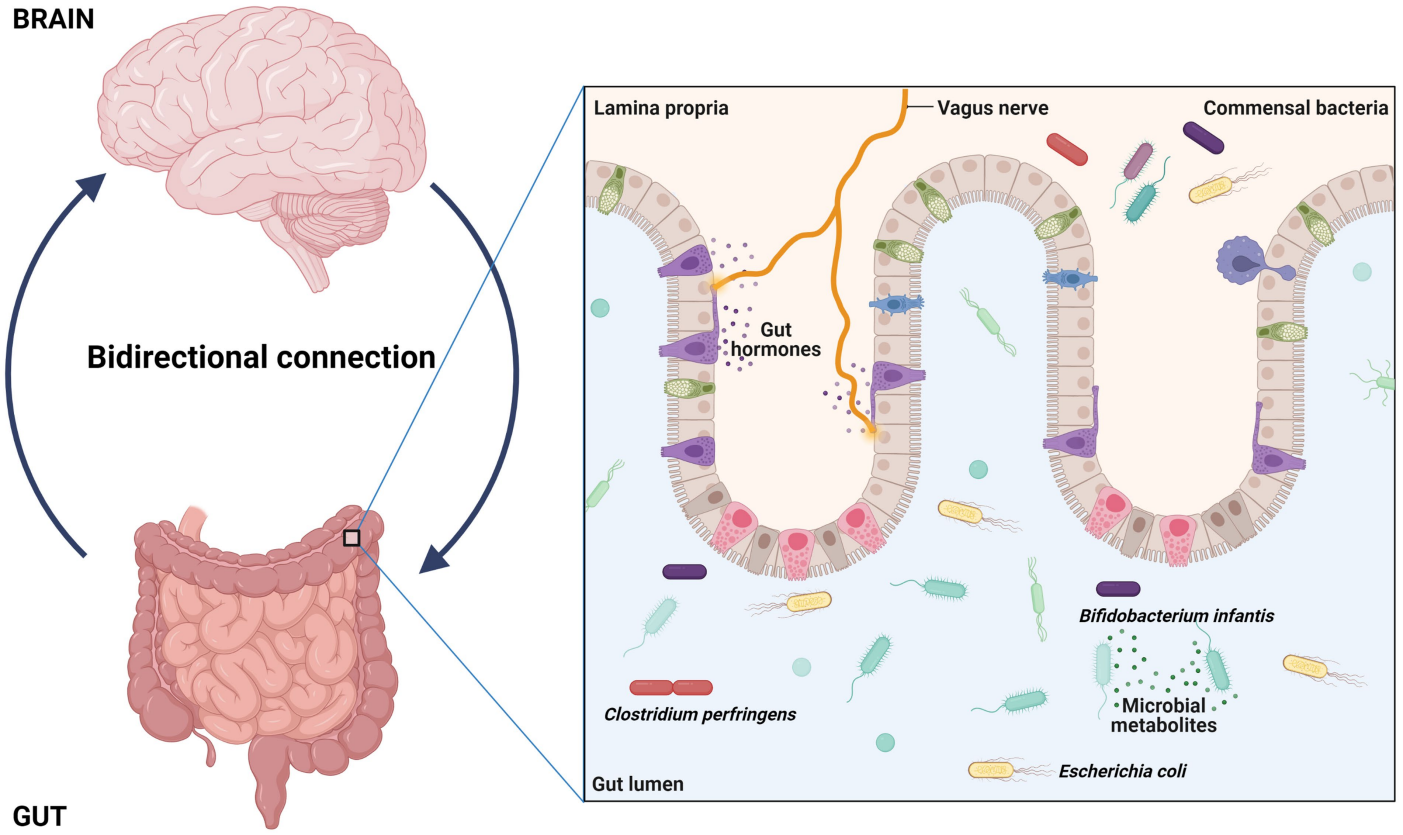
Interaction between gut and brain have potential role in regulation, development, and function of central nervous system. The gut resident bacteria and their metabolites interact with brain through a way called gut-brain axis and dysbiosis can contribute autism.

In the last years, the gut microbiota acquired an important asset for healthy status acquisition (76); as a consequence, it has been claimed as a contributor for the development of a large number of diseases (77–79). In particular, it has been established that gut-resident microbes and their metabolites can communicate with brain through the “gut-brain axis” (Figure 3); this network of bidirectional signals (involving the enteroendocrine system, metabolites, cytokines and neuroactive molecules) is crucial for the establishment of a correct gut-brain crosstalk and is able to regulate the development and functions of central nervous system through multiple mechanisms (80, 81). Thus, a perturbation of the gut microbiota (i.e., a dysbiosis) may affect these processes and play a role in the development of central nervous system diseases, including ASD (82, 83). Identify ASD specific gut microbial alterations and the mechanisms through which they can contribute to autism may provide novel diagnostic/prognostic biomarkers and new target for the improve of new therapies.

To date, gut microbiota alterations have been described in ASD mice models and patients, prompting metagenomic studies as a new frontier in autism research. In this context, Sharon et al. offered a strong evidence of the link existing between gut microbial dysbiosis and autism, demonstrating that fecal microbiota transplantation from ASD children was able to induce ASD-like behavior in germ-free mice (84). In addition, they were also able to identify differentially abundant taxa (*Clostridiaceae*, *Lactobacillales*, *Enterobacteriaceae*, and *Bacteroides*), altered expression of ASD-related genes, and decreased concentration of 5-aminovaleric acid and taurine in the ASD respect to the control group (84). Taken together, their results support the hypothesis of a role of gut microbiota in ASD and highlight some molecular alterations suggesting possible underlying mechanisms.

An altered gut microbial composition has been reported also in autistic patients compared to neurotypical children even if, to date, no specific bacterial species have been found. This was due to the existence of variable and confounding factors, such as diet, age, gender, population, and autism severity (85). Despite this variability, a decreased Bacteroidetes/Firmicutes phyla ratio, due to a decrease in the relative abundance of Bacteroidetes has been frequently reported (86). As known, Bacteroidetes are involved in polysaccharide digestion; indeed abnormal carbohydrates’ digestion in ASD patients could be due to this alteration (87). Kang et al. observed a reduction trend of the abundance of the genera *Prevotella*, *Coprococcus,* and *unclassified_Veillonellaceae* (88). Since these genera are decreased also in intestinal diseases, they suppose that ASD-related gastrointestinal symptoms may be associated to this microbial balance (88). Another study reported an increased abundance of *Clostridium bolteae* and *Clostridium cluster groups XVIII* (87). The increase of *Clostridia* was mainly related to ASD severity and worsening of symptoms probably due to the production of a neurotoxin (85, 89). In particular, Alshammari et al. reported a significantly higher abundance in ASD children than in control subjects of the *Clostridium perfringens:* this taxon produces the beta2 toxin which is associated with gastrointestinal disorders, such as diarrhoea and food poisoning (89). According to this finding, other studies reported increased level of beta2 toxin in the faeces of ASD individuals respect to the controls (88, 90). A low abundance of the genus *Akkermansia* and an increased level of *Desulfovibrio* spp. have been also reported in ASD children (91). Notably, the genus *Akkermansia* is considered beneficial while the *Desulfovibrio* harmful, as it may worsen autistic behaviors and gastrointestinal disorders (92).

In addition to such bacterial perturbations, gut fungal dysbiosis has been also reported in ASD patients. In particular, *Candida albicans*, a bacteria that produces toxic molecules as ammonia and other are associated with autism-related behavior, and *Saccharomyces cerevisiae* were most represented in autistic children gut compared to healthy controls (93, 94). Conversely, *Aspergillus versicolor* was less represented in ASD patients suggesting a possible role in the pathogenesis of ASD indeed, in addition, *S. cerevisiae* can induce an TNF increase and IL-6 production trough activation of TLR ligands (95, 96). Furthermore, the long-term antibiotic administration in ADS patients could be involved in gut microbial perturbation (97–99). It has been reported that the massive use of antibiotics could affect gut homeostasis and the exposure during pregnancy was associated to risk of develop autism in the newborn. However, oral vancomycin treatment considerably improved ASD symptoms highlighting the need for further studies on large cohorts of ASD patients in order to address the complex relationship between antibiotic exposure, gut microbiota and ASD onset (97–99). Besides the identification of specific microbial dysbiosis associated with ASD, there is growing interest in understanding how these altered taxa may contribute to disease onset and/or development. Indeed, this may lead to the development of novel therapies aimed at the microbiota manipulation, and the finding of new diagnostic/prognostic biomarkers. It has been established that dysbiosis, as previously described, can influence the gut barrier integrity, leading the “leaky gut” condition (81). In fact, an unfunctional gut barrier can increase the levels of lipopolysaccharide (LPS) in the blood stimulating the immune responses trough cytokines production, such as interferon-gamma (IFN- γ), tumor necrosis factor (TNF-ɑ) and interleukin-1beta (IL-1β); these cytokines are able to cross the blood–brain barrier (BBB), inducing systemic and CNS inflammation. In line with that, in ASD patient the serum levels of LPS were significantly increased. Furthermore, the LPS, through a lipoprotein transport mechanism, get to brain eliciting neural behavioral impairment, and neuroinflammation by triggering the Nuclear Factor Kappa B (NF-kB) signaling pathway (100, 101). In support of the “leaky gut” hypothesis, de Magistris et al. reported an altered gut permeability condition in 36.7% of ASD individuals and in 21.1% of their first-degree relatives, while only 4.8% of ordinary people showed that condition (102). In previous studies, it has been observed a significant decrease in the mRNA levels of occludin and zonulin in male mice model of idiopathic autism (BTBR mice). Occludin and zonulin are the major intestinal permeability regulators involved with the maintenance of intestinal permeability (103, 104). Others studies described that the gut permeability in ASD patients was not change, demonstrating that the disruption of the intestinal barrier is not always a hallmark of autism, but it seem be involved in ASD children with intestinal disorders (105).

As mentioned above, the interest in defining an ASD specific gut dysbiosis is driven also by the opportunity to treat ASD patients and ameliorate their clinical signs by modifying their gut microbiota. In this context, it has been reported that physical exercise positively affects the gut microbiota composition (106) which in turn reduces inflammatory responses and modulates immune and endocrine functions of enteric nervous system. As well known, the enteric nervous system is a specific entity capable of regulating the intestinal functions of mobility, secretion and mucosal transport entirely autonomously from the CNS (107). Then, this type of system can really adapt to the gut microbiota alterations, modulating all immune responses as previously mentioned. Thus, it has been speculated that the positive effects of physical exercise in ASD are due to the modulation of gut microbiota composition (108), which in turn can improve different health aspects or alleviate symptoms in ASD patients. A recent review explored the effects of exercise to improve ASD patients’ behavior through gut microbiota modifications. They found that some taxa, i.e., *Streptococcus*, *Bifidobacterium*, *Clostridium*, *Bacteroides*, and *Blautia*, are modified during exercise and may influence the gut microbiota brain axis ameliorating ASD (109). Specifically, the relative abundance of *Bacteroides* genus was significantly enriched in many patients with ASD and several scientific works reported that exercise can reduce its relative abundance (109–111). Studies have shown that high levels of *Bacteroides* affect children’s cognitive and language skills, and it is also related to the production of neurotransmitter GABA (112, 113). As well known, GABA is an amino acid neurotransmitter that functions as an important heterogeneous neurotransmitter in the central nervous system. Decreased relative abundance of *Bacteroides* can hamper pathogen invasion, reduce immune function, and promote immunity (114, 115). While *Bifidobacterium* has also been proven to be related to the production of GABA, its relative abundance in patients with ASD was found to be reduced, while some studies confirmed that physical training is able to increase its abundance. *Clostridium perfringens* was significantly increased in most of the studied autistic patients compared to the control subjects (116–118). *Clostridium perfringens* is a Gram-positive bacterium that has also been extensively studied in ASD because of its production of exotoxins and propionates, and surpisingly performing physical exercise is able to a decrease its abundance (114, 119, 120). Moreover, physical exercise has shown beneficial effects on ASD signs by ameliorating social interaction, motor skills, and communication, and reducing the severity of the disease (121). Plaza-Diaz et al. (108) reported in the Table 1 of their work an interesting list of different clinical effects related to types of physical activity, confirming the positive outcome of this non-pharmacological approach. To date, there is no clear reference for the intensity, frequency, and duration of exercise to positively perturb the gut microbiota composition and then exert all beneficial clinical effect previously described. Bonomini-Gnutzmann et al. (122) published an interesting systematic review, in which several evidences support the idea that a moderate-intensity of physical exercises, with a duration of about 30 min performed for three times at week is sufficient to increase the levels of microbial diversity, intestinal metabolites and is useful most likely to reduce the severity of clinical signs in ASD patients. It is not surprising that a structured exercise program can exert several positive effects, considering that, WHO recommended physical activity for children in order to improve cognitive, mental and physical health outcomes in children and adolescents (53). Despite, the guidelines do not include evidence concerning ASD explicitly, it highlights the positive relationship between health status and physical activity in children and adolescents with general intellectual disability (108). Despite further studies are required to clarify these connections, these aspects deserve high attention for the potential perspective in ASD treatment.

## Discussion

Autism is a neurodevelopmental disorder that alters communication and social skills. There are currently no effective therapies for ASD and the most widely used interventional approaches are mostly behavioral and educational. Numerous studies have showed that physical activity not only allows for a healthy lifestyle but also shows positive effects on social interaction which facilitates language development and improves self-esteem (14). Among different sports activities, hydrotherapy stimulate several sensory pathways through water temperature modulation, weight relief and vestibular input (42). The properties of water support active movement, amelioration of circulation and the execution motor skills. Aquatic activities also can improve social interaction and adaptive behaviors. The practice of karate stimulates the recovery of neurological capacities by increasing neuronal plasticity mediated by increasing of BDNF levels (57). In particular, the consequential rapid and controlled movements typical of karate, improve stereotypies, cognitive abilities and the communication deficit of children with ASD. Other exercise interventions, such as the minibasket, offers the possibility of practicing a group sport that harmonize their movements, improve their relationship skills and cognitive functions, allowing to channel energy into movements, and limiting the implementation of motor stereotypies (123). Furthermore, unlike other sports, in minibasket, the ball can be handled in various ways (hold in hand, pass, bounce, let slip, and fly) becoming a communication vehicle (124). Exercise interventions can be considered therapeutic tools indeed, Wang et al. (67) minibasket can be used as a complementary intervention to relieve core symptoms of ASD and to improve executive function decreasing behavioral stereotypies in preschool children with ASD. Finally, equine therapy program can be useful to improve symptoms of ASD through positive effects on cognitive and functional skills. An animal-assisted therapy correlates cognitive benefits to psychological and social aspect and was suggested as an effective treatment for ASD (72). The multisensory nature of therapeutic riding shows its stimulating effect directly associated with physical contact and the natural movement of the horse. Indeed, the act of horseback riding was perceived as a rewarding stimulus that represented higher levels of motivation and social commitment and furthermore the contact could encourage them to break away from their previous sedentary routine (125). The type of exercise is a determining variable, since the ability of an autistic patient to perform an exercise is a key factor influencing his integration into society. Several studies have shown that aerobic exercise has a significant improvement effect also because it increases the degree of contact with the outside world. Exercise load, such as quantity and intensity, is the most crucial factor during sports training (126, 127). In particular, the period and frequency of exercise significantly determine the effects of the exercise on the subject. From the point of view of the exercise period, an activity of more than 12 weeks represents a minimum time window to yield positive effects on ASD symptoms (127). From the point of view of the duration of the exercise, more than 60 min of activity allows to obtain a greater improvement. From the point of view of the frequency of the exercises, a beneficial effect can be seen with an activity carried out more than three times a week producing an effect (126, 127). The efficacy of all described treatments was mainly identified in cerebellar stimulation produced by social motivation, sedentary behaviors, and sensory stimulation. Moreover, it has been reported that physical exercise positively affects the gut microbiota brain axis ameliorating ASD. Furthermore, exercise has been reported to positively affect the gut microbiota brain axis by improving some aspects in autistic subjects (108). Furthermore, physical activity modifies the intestine microbiome leading beneficial and anti-inflammatory effects. Changes in the gut microbiota diversity and composition can translate into a reduction in inflammation and gastrointestinal symptoms as well as the modification of hundreds of metabolites (128).

The use of therapies based on physical activity in children with ASD would make it possible to stimulate different areas of the brain through external stimuli by acting on cognitive, behavioral and motor skills. In addition, these therapies act on the composition of the microbiome by modulating gastrointestinal symptoms and improving their quality of life with the reduction of the risk of developing systemic comorbidities such as obesity, gastrointestinal disorders and heart disease. Therefore, exercise represents an interesting non-pharmacological therapy for ASD. Despite the benefic effect of exercise and animal interaction the autism spectrum affection was complex and further studies are required to clarify the intricate connection between body and mind.

## Author contributions

OS and BL: conceptualization. OS, BL, AR, and CM: investigation. AR, CM, NF, IM, MI, LT, AG, and MV: data curation. OS, BL, AR, CM, RP, and VD’A: writing original draft preparation and supervision. OS, BL, AR, CM, RP, LP, and VD’A: writing review and editing. OS, BL, AR, and CM: visualization. OS and BL: project administration. All authors contributed to the article and approved the submitted version.

## References

[1] Chan JSY Deng K Yan JH. The effectiveness of physical activity interventions on communication and social functioning in autistic children and adolescents: a meta-analysis of controlled trials. Autism. (2021) 25:874–86. doi: 10.1177/1362361320977645, PMID: 33307759

[2] Lord C Brugha TS Charman T Cusack J Dumas G Frazier T. Autism spectrum disorder. Nat Rev Dis Primers. (2020) 6:5. doi: 10.1038/s41572-019-0138-4, PMID: 31949163 PMC8900942

[3] Lombardo B D’Argenio V Monda E Vitale A Caiazza M Sacchetti L. Genetic analysis resolves differential diagnosis of a familial syndromic dilated cardiomyopathy: a new case of Alström syndrome. Mol Genet Genomic Med. (2020) 8:e1260–7. doi: 10.1002/mgg3.1260, PMID: 32396277 PMC7336746

[4] Iossa S Costa V Corvino V Auletta G Barruffo L Cappellani S. Phenotypic and genetic characterization of a family carrying two Xq21.1-21.3 interstitial deletions associated with syndromic hearing loss. Mol Cytogenet. (2015) 8:18. doi: 10.1186/s13039-015-0120-0, PMID: 25821518 PMC4376344

[5] Lombardo B Ceglia C Tarsitano M Pierucci I Salvatore F Pastore L. Identification of a deletion in the NDUFS4 gene using array-comparative genomic hybridization in a patient with suspected mitochondrial respiratory disease. Gene. (2014) 535:376–9. doi: 10.1016/j.gene.2013.10.074, PMID: 24295889

[6] Ranieri A Veneruso I La Monica I Pascale MG Pastore L D’argenio V. Combined aCGH and exome sequencing analysis improves autism spectrum disorders diagnosis: a case report. Medicina. (2022) 58:1–11. doi: 10.3390/medicina58040522, PMID: 35454361 PMC9030270

[7] Falcone N Ranieri A Vitale A Pastore L Lombardo B. Identification of a De novo deletion by using A-CGH involving PLNAX2: An interesting candidate gene in psychomotor developmental delay. Medicina. (2022) 58:524. doi: 10.3390/medicina58040524, PMID: 35454363 PMC9031640

[8] Lombardo B Pagani M De Rosa A Nunziato M Migliarini S Garofalo M. D-aspartate oxidase gene duplication induces social recognition memory deficit in mice and intellectual disabilities in humans. Transl Psychiatry. (2022) 12:305–12. doi: 10.1038/s41398-022-02088-5, PMID: 35915065 PMC9343392

[9] Zebisch A Schulz E Grosso M Lombardo B Acierno G Sill H. Identification of a novel variant of epsilon-gamma-delta-beta thalassemia highlights limitations of next generation sequencing. Am J Hematol. (2015) 90:E52–4. doi: 10.1002/ajh.23913, PMID: 25488195

[10] Srinivasan SM Pescatello LS Bhat AN. Current perspectives on physical activity and exercise recommendations for children and adolescents with autism spectrum disorders. Phys Ther. (2014) 94:875–89. doi: 10.2522/ptj.20130157, PMID: 24525861 PMC4040426

[11] Fattorusso A Di Genova L Dell’isola GB Mencaroni E Esposito S. Autism spectrum disorders and the gut microbiota. Nutrients. (2019) 11:521. doi: 10.3390/nu11030521, PMID: 30823414 PMC6471505

[12] Huang J Du C Liu J Tan G. Meta-analysis on intervention effects of physical activities on children and adolescents with autism. Int J Environ Res Public Health. (2020) 17:1950. doi: 10.3390/ijerph17061950, PMID: 32192008 PMC7142971

[13] Liang X Li R Wong SHS Sum RKW Wang P Yang B. The effects of exercise interventions on executive functions in children and adolescents with autism spectrum disorder: a systematic review and meta-analysis. Sports Med. (2022) 52:75–88. doi: 10.1007/s40279-021-01545-3, PMID: 34468951

[14] Rafie F Ghasemi A Zamani Jam A Jalali S. Effect of exercise intervention on the perceptual-motor skills in adolescents with autism. J Sports Med Phys Fitness. (2017) 57:53–9. doi: 10.23736/S0022-4707.16.05919-3, PMID: 27028719

[15] Hill AP Zuckerman KE Fombonne E. Obesity and autism. Pediatrics. (2015) 136:1051–61. doi: 10.1542/peds.2015-1437, PMID: 26527551 PMC4657601

[16] Atlas of Childhood Obesity. (2019). World obes fed. Available at: http://s3-eu-west-1.amazonaws.com/wof-files/11996_Childhood_Obesity_Atlas_Report_ART_V2.

[17] Scudiero O Pero R Ranieri A Terracciano D Fimiani F Cesaro A. Childhood obesity: an overview of laboratory medicine, exercise and microbiome. Clin Chem Lab Med. (2020) 58:1385–406. doi: 10.1515/cclm-2019-0789, PMID: 31821163

[18] Sammels O Karjalainen L Dahlgren J Wentz E. Autism spectrum disorder and obesity in children: a systematic review and meta-analysis. Obes Facts. (2022) 15:305–20. doi: 10.1159/000523943, PMID: 35263756 PMC9210004

[19] Criado K Sharp W McCracken C De Vinck-Baroody O Dong L Aman M. Overweight and obese status in children with autism spectrum disorder and disruptive behavior. Autism. (2018) 22:450–9. doi: 10.1177/1362361316683888, PMID: 28325061 PMC5581311

[20] Mazurek MO Engelhardt CR. Video game use in boys with autism spectrum disorder, ADHD, or typical development. Pediatrics. (2013) 132:260–6. doi: 10.1542/peds.2012-3956 23897915

[21] Simpson M Goetz R Devlin M Goetz S Walsh B. Weight gain and antipsychotic medication: differences between antipsychotic-free and treatment periods. J Clin Psychiatry. (2001) 62:694–700. doi: 10.4088/jcp.v62n0906 11681765

[22] McDougle CJ Stigler KA Erickson CA Posey DJ. Atypical antipsychotics in children and adolescents with autistic and other pervasive developmental disorders. J Clin Psychiatry. (2008) 69:15–20. PMID: 18533764

[23] Shinawi M Sahoo T Maranda B Skinner SA Skinner C Chinault C. 11p14.1 microdeletions associated with ADHD, autism, developmental delay, and obesity. Am J Med Genet A. (2011) 155:1272–80. doi: 10.1002/ajmg.a.33878, PMID: 21567907

[24] Weiss L Shen Y Korn J Arking D Miller D Fossdal R. Association between microdeletion and microduplication at 16p11.2 and autism. N Engl J Med. (2008) 358:667–75. doi: 10.1056/NEJMoa075974, PMID: 18184952

[25] Bakalov VK Cheng C Zhou J Bondy CA. X-chromosome gene dosage and the risk of diabetes in turner syndrome. J Clin Endocrinol Metab. (2009) 94:3289–96. doi: 10.1210/jc.2009-0384, PMID: 19567529 PMC2741724

[26] Van Goor JC Massa GG. Increased incidence and prevalence of diabetes mellitus in Down’s syndrome [8]. Arch Dis Child. (1997) 77:186. doi: 10.1136/adc.77.2.183g, PMID: 9301372 PMC1717272

[27] Croonenberghs J Bosmans E Deboutte D Kenis G Maes M. Activation of the inflammatory response system in autism. Neuropsychobiology. (2002) 45:1–6. doi: 10.1159/000048665 11803234

[28] Mennitti C Ranieri A Nigro E Tripodi L Brancaccio M Ulisse J. The impact of physical exercise on obesity in a cohort of southern italian obese children: improvement in cardiovascular risk and immune system biomarkers. Int J Environ Res Public Health. (2023) 20:602. doi: 10.3390/ijerph20010602 PMC9819595 36612926

[29] Snell-Bergeon JK West NA Mayer-Davis EJ Liese AD Marcovina SM D’Agostino RB. Inflammatory markers are increased in youth with type 1 diabetes: the SEARCH case-control study. J Clin Endocrinol Metab. (2010) 95:2868–76. doi: 10.1210/jc.2009-1993, PMID: 20371668 PMC2902077

[30] Cieślak M Wojtczak A Cieślak M. Role of pro-inflammatory cytokines of pancreatic islets and prospects of elaboration of new methods for the diabetes treatment. Acta Biochim Pol. (2015) 62:15–21. doi: 10.18388/abp.2014_853, PMID: 25781159

[31] Chauhan A Chauhan V Brown WT Cohen I. Oxidative stress in autism: increased lipid peroxidation and reduced serum levels of ceruloplasmin and transferrin – the antioxidant proteins. Life Sci. (2004) 75:2539–49. doi: 10.1016/j.lfs.2004.04.038, PMID: 15363659

[32] Burstyn I Wang X Yasui Y Sithole F Zwaigenbaum L. Autism spectrum disorders and fetal hypoxia in a population-based cohort: accounting for missing exposures via estimation-maximization algorithm. BMC Med Res Methodol. (2011) 11:2. doi: 10.1186/1471-2288-11-2, PMID: 21208442 PMC3024997

[33] Onore C Careaga M Ashwood P. The role of immune dysfunction in the pathophysiology of autism. Brain Behav Immun. (2012) 26:383–92. doi: 10.1016/j.bbi.2011.08.007, PMID: 21906670 PMC3418145

[34] Schanen NC. Epigenetics of autism spectrum disorders. Hum Mol Genet. (2006) 15:R138–50. doi: 10.1093/hmg/ddl213 16987877

[35] Reynolds LC Inder TE Neil JJ Pineda RG Rogers CE. Maternal obesity and increased risk for autism and developmental delay among very preterm infants. J Perinatol. (2014) 34:688–92. doi: 10.1038/jp.2014.80, PMID: 24811227 PMC4152391

[36] Benachenhou S Etcheverry A Galarneau L Dubé J Çaku A. Implication of hypocholesterolemia in autism spectrum disorder and its associated comorbidities: a retrospective case–control study. Autism Res. (2019) 12:1860–9. doi: 10.1002/aur.2183, PMID: 31385649

[37] Petrov AM Kasimov MR Zefirov AL. Cholesterol in the pathogenesis of Alzheimer’s, Parkinson’s diseases and autism: link to synaptic dysfunction. Acta Nat. (2017) 9:26–37. doi: 10.32607/20758251-2017-9-1-26-37, PMID: 28461971 PMC5406657

[38] Ariza M Cuenca N Mauri M Jurado MA Garolera M. Neuropsychological performance of young familial hypercholesterolemia patients. Eur J Intern Med. (2016) 34:e29–31. doi: 10.1016/j.ejim.2016.05.009, PMID: 27264249

[39] Bremer E Crozier M Lloyd M. A systematic review of the behavioural outcomes following exercise interventions for children and youth with autism spectrum disorder. Autism. (2016) 20:899–915. doi: 10.1177/1362361315616002, PMID: 26823546

[40] Lang R Koegel LK Ashbaugh K Regester A Ence W Smith W. Physical exercise and individuals with autism spectrum disorders: a systematic review. Res Autism Spectr Disord. (2010) 4:565–76. doi: 10.1016/j.rasd.2010.01.006

[41] Toscano CVA Barros L Lima AB Nunes T Carvalho HM Gaspar JM. Neuroinflammation in autism spectrum disorders: exercise as a "pharmacological" tool. Neurosci Biobehav Rev. (2021) 129:63–74. doi: 10.1016/j.neubiorev.2021.07.023, PMID: 34310976

[42] Battaglia G Agrò G Cataldo P Palma A Alesi M. Influence of a specific aquatic program on social and gross motor skills in adolescents with autism spectrum disorders: three case reports. J Funct Morphol Kinesiol. (2019) 4:1–10. doi: 10.3390/jfmk4020027, PMID: 33467342 PMC7739232

[43] Mortimer R Privopoulos M Kumar S. The effectiveness of hydrotherapy in the treatment of social and behavioral aspects of children with autism spectrum disorders: a systematic review. J Multidiscip Healthc. (2014) 7:93–104. doi: 10.2147/JMDH.S55345, PMID: 24520196 PMC3917923

[44] Vodakova E Chatziioannou D Jesina O Kudlacek M. The effect of Halliwick method on aquatic skills of children with autism spectrum disorder. Int J Environ Res Public Health. (2022) 19:16250. doi: 10.3390/ijerph192316250, PMID: 36498324 PMC9738692

[45] Zanobini M Solari S. Effectiveness of the program “Acqua Mediatrice di Comunicazione” (water as a mediator of communication) on social skills, autistic behaviors and aquatic skills in ASD children. J Autism Dev Disord. (2019) 49:4134–46. doi: 10.1007/s10803-019-04128-4, PMID: 31267291

[46] Ansari S Hosseinkhanzadeh AA AdibSaber F Shojaei M Daneshfar A. The effects of aquatic versus Kata techniques training on static and dynamic balance in children with autism spectrum disorder. J Autism Dev Disord. (2021) 51:3180–6. doi: 10.1007/s10803-020-04785-w, PMID: 33206268

[47] Fragala-Pinkham MA Haley SM O’Neil ME. Group swimming and aquatic exercise programme for children with autism spectrum disorders: a pilot study. Dev Neurorehabil. (2011) 14:230–41. doi: 10.3109/17518423.2011.575438, PMID: 21732807

[48] Yanardag M Akmanoglu N Yilmaz I. The effectiveness of video prompting on teaching aquatic play skills for children with autism. Disabil Rehabil. (2013) 35:47–56. doi: 10.3109/09638288.2012.687030, PMID: 22624856

[49] Musiyenko OV Chopyk RV Kizlo NB. Influence of swimming on sensory functioning, quality of life and behavior of children with autism. Health Sport Rehab. (2020) 6:7. doi: 10.34142/HSR.2020.06.03.07

[50] Xu D Meng Y An S Meng W Li H Zhang W. Swimming exercise is a promising early intervention for autism-like behavior in Shank3 deletion rats. CNS Neurosci Ther. (2023) 29:78–90. doi: 10.1111/cns.13920, PMID: 36221783 PMC9804047

[51] Wang D Li B Wu Y Li B. The effects of maternal atrazine exposure and swimming training on spatial learning memory and hippocampal morphology in offspring male rats via PSD95/NR2B signaling pathway. Cell Mol Neurobiol. (2019) 39:1003–15. doi: 10.1007/s10571-019-00695-3 31187311 PMC11457838

[52] Alomari MA Khabour OF Alzoubi KH Alzubi MA. Forced and voluntary exercises equally improve spatial learning and memory and hippocampal BDNF levels. Behav Brain Res. (2013) 247:34–9. doi: 10.1016/j.bbr.2013.03.007, PMID: 23499703

[53] Sefen JAN Al-Salmi S Shaikh Z AlMulhem JT Rajab E Fredericks S. Beneficial use and potential effectiveness of physical activity in managing autism spectrum disorder. Front Behav Neurosci. (2020) 14:1–8. doi: 10.3389/fnbeh.2020.587560, PMID: 33192368 PMC7642468

[54] Bahrami F Movahedi A Marandi SM Sorensen C. The effect of karate techniques training on communication deficit of children with autism spectrum disorders. J Autism Dev Disord. (2016) 46:978–86. doi: 10.1007/s10803-015-2643-y, PMID: 26577688

[55] Kasarpalkar NJ Kothari ST Dave UP. Brain-derived neurotrophic factor in children with autism spectrum disorder. Ann Neurosci. (2014) 21:129–33. doi: 10.5214/ans.0972.7531.210403, PMID: 25452672 PMC4248479

[56] Bhattacharya P Chatterjee S Mondal S. Effect of karate on neurocognitive physiology: a focused review. Neurol India. (2022) 70:11–8. doi: 10.4103/0028-3886.338688, PMID: 35263847

[57] Miranda M Morici JF Zanoni MB Bekinschtein P. Brain-derived Neurotrophic factor: a key molecule for memory in the healthy and the pathological brain. Front Cell Neurosci. (2019) 13:1–25. doi: 10.3389/fncel.2019.00363, PMID: 31440144 PMC6692714

[58] Chaturvedi P Singh A Tiwari V Thacker A. Brain-derived neurotrophic factor levels in acute stroke and its clinical implications. Brain Circ. (2020) 6:185–90. doi: 10.4103/bc.bc_23_20, PMID: 33210043 PMC7646383

[59] Barbosa AG Pratesi R Paz GSC dos Santos MAAL Uenishi RH Nakano EY. Assessment of BDNF serum levels as a diagnostic marker in children with autism spectrum disorder. Sci Rep. (2020) 10:17348–7. doi: 10.1038/s41598-020-74239-x, PMID: 33060610 PMC7566481

[60] Walsh J Tschakovsky M. Exercise and circulating BDNF: mechanisms of release and implications for the design of exercise interventions. Appl Physiol Nutr Metab. (2018) 43:1095–104. doi: 10.1139/apnm-2018-0192, PMID: 29775542

[61] Yang S Liu Z Xiong X Cai K Zhu L Dong X. Effects of mini-basketball training program on social communication impairment and executive control network in preschool children with autism spectrum disorder. Int J Environ Res Public Health. (2021) 18:5132. doi: 10.3390/ijerph18105132, PMID: 34066139 PMC8150962

[62] Cai KL Wang JG Liu ZM Zhu LN Xiong X Klich S. Mini-basketball training program improves physical fitness and social communication in preschool children with autism spectrum disorders. J Hum Kinet. (2020) 73:267–78. doi: 10.2478/hukin-2020-0007, PMID: 32774558 PMC7386133

[63] Fatemi SH. Cerebellar pathology in autism. Essent Cerebellum Cerebellar Disord A Primer Grad Stud. (2016) 11:539–43. doi: 10.1007/978-3-319-24551-5_72

[64] Manto M Bower JM Conforto AB Delgado-García JM da Guarda SNF Gerwig M. Cerebellar function and cognition. PMC. (2015) 11:457–87. doi: 10.1007/s12311-011-0331-9.Consensus

[65] Bamidis PD Vivas AB Styliadis C Frantzidis C Klados M Schlee W. A review of physical and cognitive interventions in aging. Neurosci Biobehav Rev. (2014) 44:206–20. doi: 10.1016/j.neubiorev.2014.03.019 24705268

[66] Jeon YK Ha CH. The effect of exercise intensity on brain derived neurotrophic factor and memory in adolescents. Environ Health Prev Med. (2017) 22:27–6. doi: 10.1186/s12199-017-0643-6, PMID: 29165142 PMC5664787

[67] Wang JG Cai KL Liu ZM Herold F Zou L Zhu LN. Effects of mini-basketball training program on executive functions and core symptoms among preschool children with autism spectrum disorders. Brain Sci. (2020) 10:1–14. doi: 10.3390/brainsci10050263, PMID: 32365853 PMC7287705

[68] Borgi M Loliva D Cerino S Chiarotti F Venerosi A Bramini M. Effectiveness of a standardized equine-assisted therapy program for children with autism spectrum disorder. J Autism Dev Disord. (2016) 46:1–9. doi: 10.1007/s10803-015-2530-6, PMID: 26210515

[69] Zhao M Chen S You Y Wang Y Zhang Y. Effects of a therapeutic horseback riding program on social interaction and communication in children with autism. Int J Environ Res Public Health. (2021) 18:1–11. doi: 10.3390/ijerph18052656, PMID: 33800787 PMC7967314

[70] Srinivasan SM Cavagnino DT Bhat AN. Effects of equine therapy on individuals with autism spectrum disorder: a systematic review. Rev J Autism Dev Disord. (2018) 5:156–75. doi: 10.1007/s40489-018-0130-z, PMID: 30319932 PMC6178825

[71] Anderson S Meints K. Brief report: the effects of equine-assisted activities on the social functioning in children and adolescents with autism spectrum disorder. J Autism Dev Disord. (2016) 46:3344–52. doi: 10.1007/s10803-016-2869-3, PMID: 27457363 PMC5040734

[72] O’Haire ME. Animal-assisted intervention for autism spectrum disorder: a systematic literature review. J Autism Dev Disord. (2013) 43:1606–22. doi: 10.1007/s10803-012-1707-5, PMID: 23124442

[73] Chen S Zhang Y Zhao M Du X Wang Y Liu X. Effects of therapeutic horseback-riding program on social and communication skills in children with autism spectrum disorder: a systematic review and meta-analysis. Int J Environ Res Public Health. (2022) 19:14449. doi: 10.3390/ijerph192114449, PMID: 36361327 PMC9655675

[74] McDaniel Peters BC Wood W. Autism and equine-assisted interventions: a systematic mapping review. J Autism Dev Disord. (2017) 47:3220–42. doi: 10.1007/s10803-017-3219-9, PMID: 28733851

[75] Llambias C Magill-Evans J Smith V Warren S. Equine-assisted occupational therapy: increasing engagement for children with autism spectrum disorder. Am J Occup Ther. (2016) 70:7006220040p1–9. doi: 10.5014/ajot.2016.020701, PMID: 27767943

[76] D’Argenio V. Human microbiome acquisition and bioinformatic challenges in metagenomic studies. Int J Mol Sci. (2018) 19:1–12. doi: 10.3390/ijms19020383, PMID: 29382070 PMC5855605

[77] Iaffaldano L Granata I Pagliuca C Esposito MV Casaburi G Salerno G. Oropharyngeal microbiome evaluation highlights Neisseria abundance in active celiac patients. Sci Rep. (2018) 8:11047–10. doi: 10.1038/s41598-018-29443-1, PMID: 30038321 PMC6056421

[78] Nardelli C Granata I D’argenio V Tramontano S Compare D Guarracino MR. Characterization of the duodenal mucosal microbiome in obese adult subjects by 16s rRNA sequencing. Microorganisms. (2020) 8:1–13. doi: 10.3390/microorganisms8040485, PMID: 32235377 PMC7232320

[79] D’Argenio V Veneruso I Gong C Cecarini V Bonfili L Eleuteri AM. Gut microbiome and Mycobiome alterations in an in vivo model of Alzheimer’s disease. Genes (Basel). (2022) 13:1564. doi: 10.3390/genes13091564, PMID: 36140732 PMC9498768

[80] Schmidt TSB Hayward MR Coelho LP Li SS Costea PI Voigt AY. Extensive transmission of microbes along the gastrointestinal tract. elife. (2019) 8:e42693. doi: 10.7554/eLife.42693, PMID: 30747106 PMC6424576

[81] Yitik Tonkaz G Esin IS Turan B Uslu H Dursun OB. Determinants of leaky gut and gut microbiota differences in children with autism spectrum disorder and their siblings. J Autism Dev Disord. (2022) 53:2703–16. doi: 10.1007/s10803-022-05540-z, PMID: 35441922

[82] Yang Y Tian J Yang B. Targeting gut microbiome: a novel and potential therapy for autism. Life Sci. (2018) 194:111–9. doi: 10.1016/j.lfs.2017.12.027, PMID: 29277311

[83] D’Argenio V Sarnataro D. Microbiome influence in the pathogenesis of prion and Alzheimer’s diseases. Int J Mol Sci. (2019) 20:4704. doi: 10.3390/ijms20194704, PMID: 31547531 PMC6801937

[84] Sharon G Cruz N Kang DW Gandal MJ Wang B Kim YM. Human gut microbiota from autism spectrum disorder promote behavioral symptoms in mice. Cells. (2019) 177:1600–1618.e17. doi: 10.1016/j.cell.2019.05.004, PMID: 31150625 PMC6993574

[85] Sivamaruthi B Suganthy N Kesika P Chaiyasut C. The role of microbiome, dietary supplements, and probiotics in autism spectrum disorder. Int J Environ Res Public Health. (2020) 17:2647. doi: 10.3390/ijerph17082647, PMID: 32290635 PMC7215504

[86] Settanni CR Bibbò S Ianiro G Rinninella E Cintoni M Mele MC. Gastrointestinal involvement of autism spectrum disorder: focus on gut microbiota. Expert Rev Gastroenterol Hepatol. (2021) 15:599–622. doi: 10.1080/17474124.2021.1869938, PMID: 33356668

[87] Pulikkan J Mazumder A Grace T. Role of the gut microbiome in autism spectrum disorders. Adv Exp Med Biol. (2019) 1118:253–69. doi: 10.1007/978-3-030-05542-4_13 30747427

[88] Kang DW Park JG Ilhan ZE Wallstrom G LaBaer J Adams JB. Reduced incidence of Prevotella and other fermenters in intestinal microflora of autistic children. PLoS One. (2013) 8:e68322. doi: 10.1371/journal.pone.0068322, PMID: 23844187 PMC3700858

[89] Alshammari MK AlKhulaifi MM Al Farraj DA Somily AM Albarrag AM. Incidence of Clostridium perfringens and its toxin genes in the gut of children with autism spectrum disorder. Anaerobe. (2020) 61:102114–1. doi: 10.1016/j.anaerobe.2019.102114, PMID: 31704282

[90] Fisher DJ Miyamoto K Harrison B Akimoto S Sarker MR McClane BA. Association of beta2 toxin production with Clostridium perfringens type a human gastrointestinal disease isolates carrying a plasmid enterotoxin gene. Mol Microbiol. (2005) 56:747–62. doi: 10.1111/j.1365-2958.2005.04573.x, PMID: 15819629

[91] Wang L Conlon MA Christophersen CT Sorich MJ Angley MT. Gastrointestinal microbiota and metabolite biomarkers in children with autism spectrum disorders. Biomark Med. (2014) 8:331–44. doi: 10.2217/bmm.14.12 24712423

[92] Kovtun AS Averina OV Alekseeva MG Danilenko VN. Antibiotic resistance genes in the gut microbiota of children with autistic spectrum disorder as possible predictors of the disease. Microb Drug Resist. (2020) 26:1307–20. doi: 10.1089/mdr.2019.0325, PMID: 31916894

[93] Herman A Herman AP. Could Candida overgrowth be involved in the pathophysiology of autism? J Clin Med. (2022) 11:442. doi: 10.3390/jcm11020442, PMID: 35054136 PMC8778531

[94] Kantarcioglu A Kiraz N Aydin A. Microbiota-gut-brain axis: yeast species isolated from stool samples of children with suspected or diagnosed autism spectrum disorders and in vitro susceptibility against nystatin and fluconazole. Mycopathologia. (2016) 181:1–7. doi: 10.1007/s11046-015-9949-3, PMID: 26442855

[95] Mehra A Arora G Sahni G Kaur M Singh H Singh B. Gut microbiota and autism spectrum disorder: from pathogenesis to potential therapeutic perspectives. J Tradit Complement Med. (2022) 13:135–49. doi: 10.1016/j.jtcme.2022.03.001, PMID: 36970459 PMC10037072

[96] Zou R Wang Y Duan M Guo M Zhang Q Zheng H. Dysbiosis of gut fungal microbiota in children with autism spectrum disorders. J Autism Dev Disord. (2021) 51:267–75. doi: 10.1007/s10803-020-04543-y, PMID: 32447559

[97] Almasri J Barazi A King KS Walther-Antonio MRS Wang Z Murad MH. Peripartum antibiotics exposure and the risk of autoimmune and autism disorders in the offspring. Avicenna J Med. (2021) 11:118–25. doi: 10.1055/s-0041-1732485, PMID: 34646788 PMC8500092

[98] Ristori MV Quagliariello A Reddel S Ianiro G Vicari S Gasbarrini A. Autism, gastrointestinal symptoms and modulation of gut microbiota by nutritional interventions. Nutrients. (2019) 11:1–21. doi: 10.3390/nu11112812, PMID: 31752095 PMC6893818

[99] Yassour M Vatanen T Siljander H Hämäläinen A Härkönen T Ryhänen S. Natural history of the infant gut microbiome and impact of antibiotic treatment on bacterial strain diversity and stability. Sci Transl Med. (2016) 8:343ra81. doi: 10.1126/scitranslmed.aad0917, PMID: 27306663 PMC5032909

[100] Vargas-Caraveo A Sayd A Maus SR Caso JR Madrigal JLM García-Bueno B. Lipopolysaccharide enters the rat brain by a lipoprotein-mediated transport mechanism in physiological conditions. Sci Rep. (2017) 7:1–15. doi: 10.1038/s41598-017-13302-6 29030613 PMC5640642

[101] Zhao J Bi W Xiao S Lan X Cheng X Zhang J. Neuroinflammation induced by lipopolysaccharide causes cognitive impairment in mice. Sci Rep. (2019) 9:5790–12. doi: 10.1038/s41598-019-42286-8, PMID: 30962497 PMC6453933

[102] De Magistris L Familiari V Pascotto A Sapone A Frolli A Iardino P. Alterations of the intestinal barrier in patients with autism spectrum disorders and in their first-degree relatives. J Pediatr Gastroenterol Nutr. (2010) 51:418–24. doi: 10.1097/MPG.0b013e3181dcc4a5, PMID: 20683204

[103] Sochaczewska D Ziętek M Dołęgowska B Kordek A Szczuko M. Implications of indirect biomarkers of intestinal permeability in the stools of newborns and infants with perinatal risk factors for intestinal colonization disorders and infant feeding patterns. Nutrients. (2022) 14:2224. doi: 10.3390/nu14112224, PMID: 35684026 PMC9182768

[104] Paparo L Tripodi L Bruno C Pisapia L Damiano C Pastore L. Protective action of Bacillus clausii probiotic strains in an in vitro model of Rotavirus infection. Sci Rep. (2020) 10:1–10. doi: 10.1038/s41598-020-69533-7 32724066 PMC7387476

[105] Fowlie G Cohen N Ming X. The perturbance of microbiome and gut-brain axis in autism spectrum disorders. Int J Mol Sci. (2018) 19:2251. doi: 10.3390/ijms19082251, PMID: 30071612 PMC6121241

[106] Koutouratsas T Gazouli M Philippou A Koutsilieris M Kolios G. Role of exercise in preventing and restoring gut dysbiosis in patients with inflammatory bowel diseases: a review. World J Gastroenterol. (2021) 27:5037–46. doi: 10.3748/wjg.v27.i30.5037, PMID: 34497433 PMC8384738

[107] Sharma M Prakash J Yadav P Srivastava K Chatterjee K. Gut-brain axis: Synergistic approach. Ind Psychiatry J. (2021) 30:S297–300. doi: 10.4103/0972-6748.328835, PMID: 34908715 PMC8611583

[108] Plaza-Diaz J Radar AM Baig AT Leyba MF Costabel MM Zavala-Crichton JP. Physical activity, gut microbiota, and genetic background for children and adolescents with autism spectrum disorder. Child Aust. (2022) 9:1–22. doi: 10.3390/children9121834, PMID: 36553278 PMC9777368

[109] Xue Y An S Qiu W Zhang W Fu L Zhen Z. Exercise changes gut microbiota: a new idea to explain that exercise improves autism. Int J Sports Med. (2023) 44:473–83. doi: 10.1055/a-2018-2477, PMID: 36690029

[110] Zurita MF Cárdenas PA Sandoval ME Peña MC Fornasini M Flores N. Analysis of gut microbiome, nutrition and immune status in autism spectrum disorder: a case-control study in Ecuador. Gut Microbes. (2020) 11:453–64. doi: 10.1080/19490976.2019.1662260, PMID: 31530087 PMC7524316

[111] Dan Z Mao X Liu Q Guo M Zhuang Y Liu Z. Altered gut microbial profile is associated with abnormal metabolism activity of Autism Spectrum Disorder. Gut Microbes. (2020) 11:1246–67. doi: 10.1080/19490976.2020.1747329, PMID: 32312186 PMC7524265

[112] Tamana SK Tun HM Konya T Chari RS Field CJ Guttman DS. Bacteroides-dominant gut microbiome of late infancy is associated with enhanced neurodevelopment. Gut Microbes. (2021) 13:1–17. doi: 10.1080/19490976.2021.1930875, PMID: 34132157 PMC8210878

[113] Strandwitz P Kim KH Terekhova D Liu JK Sharma A Levering J. GABA-modulating bacteria of the human gut microbiota. Nat Microbiol. (2019) 4:396–403. doi: 10.1038/s41564-018-0307-3, PMID: 30531975 PMC6384127

[114] Karl JP Margolis LM Madslien EH Murphy NE Castellani JW Gundersen Y. Changes in intestinal microbiota composition and metabolism coincide with increased intestinal permeability in young adults under prolonged physiological stress. Am J Physiol Gastrointest Liver Physiol. (2017) 312:G559–71. doi: 10.1152/ajpgi.00066.2017, PMID: 28336545

[115] Barrett E Ross RP O'Toole PW Fitzgerald GF Stanton C. γ-Aminobutyric acid production by culturable bacteria from the human intestine. J Appl Microbiol. (2012) 113:411–7. doi: 10.1111/j.1365-2672.2012.05344.x, PMID: 22612585

[116] Luna RA Oezguen N Balderas M Venkatachalam A Runge JK Versalovic J. Distinct microbiome-neuroimmune signatures correlate with functional abdominal pain in children with autism spectrum disorder. Cell Mol Gastroenterol Hepatol. (2016) 3:218–30. doi: 10.1016/j.jcmgh.2016.11.008, PMID: 28275689 PMC5331780

[117] De Angelis M Piccolo M Vannini L Siragusa S De Giacomo A Serrazzanetti DI. Fecal microbiota and metabolome of children with autism and pervasive developmental disorder not otherwise specified. PLoS One. (2013) 8:e76993. doi: 10.1371/journal.pone.0076993, PMID: 24130822 PMC3793965

[118] Chua HH Chou HC Tung YL Chiang BL Liao CC Liu HH. Intestinal dysbiosis featuring abundance of Ruminococcus gnavus associates with allergic diseases in infants. Gastroenterology. (2018) 154:154–67. doi: 10.1053/j.gastro.2017.09.006, PMID: 28912020

[119] Tabone M Bressa C García-Merino JA Moreno-Pérez D Van EC Castelli FA. The effect of acute moderate-intensity exercise on the serum and fecal metabolomes and the gut microbiota of cross-country endurance athletes. Sci Rep. (2021) 11:3558. doi: 10.1038/s41598-021-82947-1, PMID: 33574413 PMC7878499

[120] Quiroga R Nistal E Estébanez B Porras D Juárez-Fernández M Martínez-Flórez S. Exercise training modulates the gut microbiota profile and impairs inflammatory signaling pathways in obese children. Exp Mol Med. (2020) 52:1048–61. doi: 10.1038/s12276-020-0459-0, PMID: 32624568 PMC8080668

[121] Jia S Guo C Li S Zhou X Wang X Wang Q. The effect of physical exercise on disordered social communication in individuals with autism Spectrum disorder: a systematic review and meta-analysis of randomized controlled trials. Front Pediatr. (2023) 11:1193648. doi: 10.3389/fped.2023.1193648, PMID: 37456563 PMC10347521

[122] Bonomini-Gnutzmann R Plaza-Díaz J Jorquera-Aguilera C Rodríguez-Rodríguez A Rodríguez-Rodríguez F. Effect of intensity and duration of exercise on gut microbiota in humans: a systematic review. Int J Environ Res Public Health. (2022) 19:9518. doi: 10.3390/ijerph19159518 35954878 PMC9368618

[123] Fotrousi F Bagherly J Ghasemi A. The compensatory impact of mini-basketball skills on the Progress of fundamental movements in children. Procedia Soc Behav Sci. (2012) 46:5206–10. doi: 10.1016/j.sbspro.2012.06.410

[124] Lambert JM Copeland BA Karp EL Finley CI Houchins-Juarez NJ Ledford JR. Chaining functional basketball sequences (with embedded conditional discriminations) in an adolescent with autism. Behav Anal Pract. (2016) 9:199–210. doi: 10.1007/s40617-016-0125-0, PMID: 27622126 PMC4999364

[125] Bass MM Duchowny CA Llabre MM. The effect of therapeutic horseback riding on social functioning in children with autism. J Autism Dev Disord. (2009) 39:1261–7. doi: 10.1007/s10803-009-0734-3, PMID: 19350376

[126] Zhao M Chen S. The effects of structured physical activity program on social interaction and communication for children with autism. Biomed Res Int. (2018) 2018:1825046. doi: 10.1155/2018/1825046, PMID: 29568743 PMC5820623

[127] Ji YQ Tian H Zheng ZY Ye ZY Ye Q. Effectiveness of exercise intervention on improving fundamental motor skills in children with autism spectrum disorder: a systematic review and meta-analysis. Front Psychiatry. (2023) 14:1132074. doi: 10.3389/fpsyt.2023.1132074, PMID: 37377477 PMC10291092

[128] Clauss M Gérard P Mosca A Leclerc M. Interplay between exercise and gut microbiome in the context of human health and performance. Front Nutr. (2021) 8:637010. doi: 10.3389/fnut.2021.637010, PMID: 34179053 PMC8222532

[129] Devnani P Hegde A. Autism and sleep disorders. J Pediatr Neurosci. (2015) 10:304–7. doi: 10.4103/1817-1745.174438, PMID: 26962332 PMC4770638

[130] Veatch OJ Maxwell-Horn AC Malow BA. Sleep in autism spectrum disorders. Curr Sleep Med Rep. (2015) 1:131–40. doi: 10.1007/s40675-015-0012-1, PMID: 26046012 PMC4450800

[131] Dhaliwal KK Orsso CE Richard C Haqq AM Zwaigenbaum L. Risk factors for unhealthy weight gain and obesity among children with autism spectrum disorder. Int J Mol Sci. (2019) 20:3285. doi: 10.3390/ijms20133285, PMID: 31277383 PMC6650879

[132] Esposito CM Buoli M Ciappolino V Agostoni C Brambilla P. The role of cholesterol and fatty acids in the etiology and diagnosis of autism spectrum disorders. Int J Mol Sci. (2021) 22:3550. doi: 10.3390/ijms22073550, PMID: 33805572 PMC8036564

